# General practice based psychosocial interventions for supporting carers of people with dementia or stroke: a systematic review 

**DOI:** 10.1186/s12875-015-0399-2

**Published:** 2016-01-15

**Authors:** Nan Greenwood, Ferruccio Pelone, Anne-Marie Hassenkamp

**Affiliations:** Faculty of Health, Social Care and Education, Kingston University and St George’s, University of London, London, UK

**Keywords:** Carer, Caregiver, General practice, Stroke, Dementia, Psychosocial intervention

## Abstract

**Background:**

Particularly with ageing populations, dementia and stroke and their resultant disability are worldwide concerns. Much of the support for people with these conditions comes from unpaid carers or caregivers. The carers’ role is often challenging and carers themselves may need support. General practice is often the first point of contact for people with these conditions and their carers, making it potentially an important source of support.

This systematic review therefore synthesised the available evidence for the impact of supportive interventions for carers provided in general practice.

**Methods:**

PRISMA guidelines were adopted and the following databases were searched: MEDLINE; EMBASE; the Cochrane Library; PsycINFO; CINAHL Plus; Applied Social Sciences Index and Abstracts and Healthcare Management Information Consortium.

**Results:**

Two thousand four hundred eighty nine results were identified. Four studies, involving 447 carers, fitted the inclusion criteria. Three of these came from the United States of America. None investigated supportive interventions for carers of people with stroke. Primarily by the provision of information and educational materials, the interventions focussed on improving carer mental health, dementia knowledge, caregiving competence and reducing burden, difficulties and frustrations. Overall the evidence suggests that these interventions may improve carer well-being and emotional health but the impact on physical health and social variables was less clear. However, the diversity of the carer outcomes and the measures used means that the findings must be viewed with caution.

**Conclusions:**

Unpaid carers pay an essential role in caring for people with stroke and dementia and the dearth of literature investigating the impact of supportive interventions for these carers of is surprising. The available evidence suggests that it may be possible to offer support for these carers in general practice but future research should consider focussing on the same outcome measures in order to allow comparisons across interventions.

**Electronic supplementary material:**

The online version of this article (doi:10.1186/s12875-015-0399-2) contains supplementary material, which is available to authorized users.

## Background

Stroke and dementia result in both long and short term disability [1, 2] and worldwide both are major health and social care issues. However, with ageing populations and the move away from institutional care, the numbers of people living in the community with dementia [3] or post-stroke disability [4] are rising. Although clearly very different, stroke and dementia are both long-term conditions whose impact goes far beyond the individuals with the condition.

Stroke is recognised as the major cause of complex long-term disability in adults worldwide [5]. After hospitalisation and rehabilitation approximately 80 % of stroke survivors return home with much of their care provided by families [6, 7]. However, undertaking this role is known to have negative consequences for these unpaid carers [8, 9]. The picture for dementia is very similar to stroke with unpaid family carers providing most of the support for people with dementia living at home [3, 10], with similar challenges faced by carers across countries and care systems [11]. However, research shows that carers of people with dementia frequently have poorer physical and emotional health than carers of people with other long-term conditions [12, 13].

Over the past decade there have been several studies evaluating and reviewing the evidence for the impact of support interventions for family carers of community dwelling relatives. Existing reviews of interventions intended to support family carers have focused mostly on dementia [14–17] with relatively few looking at interventions for carers of people with stroke [4, 18]. By and large these reviews have concentrated on particular types of interventions (e.g. information provision, psychosocial or educational interventions), or particular outcomes (e.g. burden or quality of life). Overall the evidence for the effectiveness of these interventions is mixed but some studies suggest a positive impact of, for example, information provision [18] and psycho-education on, for example, carer depression and problem-solving [19].

General practice teams are often the first point of contact for services for carers making them well placed to recognise and support carers of people with all health conditions including stroke and dementia [20–22]. Evidence for stroke, for example, suggests that both stroke survivors and their carers want regular contact with their general practitioners (GPs) [23] whilst some later research suggests that GPs and other members of primary care teams believe they have an important role to play in supporting carers but lack time and resources to do so [24].

However, to the best of our knowledge, there is no systematic review looking at interventions offered in general practice to support carers. Therefore, the aim of this systematic review is to identify, appraise and summarise all the published evidence on general practice based interventions to support carers of people with stroke or dementia.

This review was based upon the following specific questions:

1. What interventions are offered by general practice to support carers of people with dementia or stroke?
2. What are the most effective general practice based interventions to support carers of people with dementia or stroke?
3. What are the implications for future research in this area?

## Methods

The review followed PRISMA guidelines [25], and the protocol was registered with PROSPERO the international prospective register of systematic reviews at the Centre for Reviews and Dissemination, University of York; registration number CRD42015016056 [26].

### Search strategy

The following electronic databases were searched: MEDLINE; EMBASE; the Cochrane Library; PsycINFO; CINAHL Plus; Applied Social Sciences Index and Abstracts; Healthcare Management Information Consortium. A Medline search strategy (Table 1) was developed and was modified where necessary to run on the other databases.

**Table 1:** MEDLINE (OVID) search strategy: inception to 2014 (updated in July 2015)

ID	Concept	Search (*Hits = n*)
1	General practice	(General practice$ or General practitioner$ or GPs).tw. (40707)
2		(family practice$ or family practitioner$ or family physician$ family medicine$).tw. (4097)
3		(district nurse$ or practice nurse$).tw. (3976)
4		(community NEAR/3 health).tw. (18269)
5		(community NEAR/3 care).tw. (8627)
6		(community NEAR/3 services).tw. (4750)
7		exp Primary Health Care/ (62263)
8		exp Family Practice/ (32389)
9		exp Physicians, Family/ (9205)
10		exp General Practitioners/ (2116)
11		exp General Practice/ (36336)
12		1 or 2 or 3 or 4 or 5 or 6 or 7 or 8 or 9 or 10 or 11 (146870)
13	Carers	(informal NEAR/5 (care-giver$ or caregiv$ or carer$)).tw. (1718)
14		(family NEAR/5 (care-giver$ or caregiv$ or carer$)).tw. (5332)
15		(spouse$ NEAR/5 (care-giver$ or caregiv$ or carer$)).tw. (602)
16		(relative$ NEAR/5 (care-giver$ or caregiv$ or carer$)).tw. (829)
17		(parent$ NEAR/5 (care-giver$ or caregiv$ or carer$)).tw. (2222)
18		(brother$ NEAR/5 (care-giver$ or caregiv$ or carer$)).tw. (5)
19		(sister$ NEAR/5 (care-giver$ or caregiv$ or carer$)).tw. (5)
20		exp Caregivers/ (*19868*)
21		13 or 14 or 15 or 16 or 17 or 18 or 19 or 20 (23437)
22	Stroke & Dementia	exp Alzheimer Disease/ (53196)
23		exp Lewy Body Disease/ (2092)
24		exp Dementia, Vascular/ or exp Dementia, Multi-Infarct/ or exp Dementia/ or exp Frontotemporal Dementia/ (89101)
25		dement$.tw. (50486)
26		exp Stroke/ (77056)
27		Cerebrovascular Disorders/ (12999)
28		(cva or cerebrovascular or cerebral vascular or stroke$ or brain vasc$).tw. (130119)
29		22 or 23 or 24 or 25 or 26 or 27 or 28 (248841)
30	General practice based interventions for carers of people with stroke or dementia	12 and 21 and 29 (491)
31		limit 30 to (English language and humans) (445)

Searches were from database inception to December 2014 (an updated search was undertaken in July 2015) and was limited to articles written in English.

The electronic database search was supplemented by three strategies: 1) manual screening of the reference lists of any previous similar reviews; 2) manual screening of the reference lists of included studies; 3) and hand-searching of two relevant journals focusing on general practice and dementia/stroke for relevant publications over the last 10 years.

### Inclusion criteria and study selection

Studies of any design were considered if they fitted the following inclusion criteria:

- Population: Carers of people with dementia (including relatives, spouses and friends) and/or carers of people with stroke/stroke survivors (including relatives, spouses and friends).
- Intervention: Any non-pharmacological intervention delivered by healthcare providers from general practice (e.g. GPs, practice nurses) aimed to improve carer outcomes: e.g. educational interventions, skills training interventions, psychological interventions, counselling and information provision.
- Setting: General practice, which includes healthcare providers who are ‘primarily responsible for the provision of comprehensive and continuing care to every individual seeking medical care irrespective of age, sex and illness’ [27].
- Outcomes: e.g. carer mental health (including psychological well-being, depression, and anxiety), carer competency (including coping strategies, knowledge of dementia, self-efficacy and responses to disruptive behaviour) and carer burden.
- Study characteristics: Studies were included if they reported empirical findings and outcomes regardless of their study design. This included quantitative, qualitative and mixed methods studies

Articles were excluded if they did not fulfil one or more inclusion criteria or if they were literature reviews, case series, case studies, commentaries, not peer-review articles and/or unpublished. After duplicate removal, a two-step selection process was performed: a) Title and abstract screening; 2) Full-text screening. All records identified were independently reviewed by two reviewers (NG and AMH). Abstracts were assessed and full texts of studies not excluded at this stage, were retrieved for further evaluation. Discrepancies were resolved by discussion or when necessary were decided by the third reviewer (FP).

### Data extraction

Two reviewers (NG and AMH) independently extracted the data from selected papers, with any disagreement resolved by the third author (FP). A checklist was used to extract the following information from the selected papers:

- General information, for example, year of publication, reported study type, research objective/aim(s)
- Descriptive information, for example, description of the intervention (including setting, time period, frequency, and intervention duration); study population characteristics (including relevant demographic characteristics, inclusion/exclusion criteria, participant numbers); outcomes measured and data analysis
- Study quality
- Results including findings and the reported discussion & conclusions.

### Assessment of methodological quality

The quality of included studies was appraised using the quality checklists for quantitative and qualitative studies [28]. The broad nature of the quality assessment allows a range of methodologies to be assessed. For the quantitative studies, 14 items were scored depending on the extent to which the specific criteria were met (“yes” = 2, “partial” = 1, “no” = 0). Items not applicable to a particular study design were marked “n/a” and were excluded from the calculation of the summary score.

### Data synthesis

A meta-analysis was not performed due to the heterogeneity of the included studies. Therefore, narrative synthesis was conducted.

## Results

**Fig. 1:**
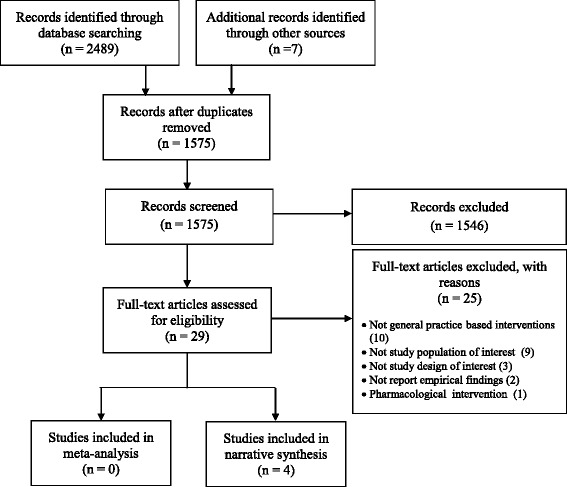
PRISMA flow diagram for the inclusion of studies

The electronic searches retrieved a total of 2489 results. As shown in Fig. 1, 29 full-text articles were reviewed against the inclusion criteria leading to the selection of four for narrative synthesis. Additional file 1 provides references list of the 25 excluded studies in the last stage of the screening process.

All selected studies evaluated general practice based interventions for carers of people with dementia and none investigated support for carers of people with stroke (Table 2). Three studies included carers of people with Alzheimer’s disease and related dementias [29–31]. The remaining study [32] included carers of people with dementia and carers of people with a mixture of other chronic disorders (e.g. heart disease and musculoskeletal disorders). Carer participant sample sizes ranged from 31 [31] to 164 [29]. In total, 447 carer participants were included in the four studies. The maximum reported attrition rate was 57 % [29]. Carers were mostly female with a mean age of between 61 and 72 years.

**Table 2:** Summary characteristics of included papers

Reference	Country Setting	Methods and intervention	Carer participants			
			1) Intervention group	Number* (Attrition %)	Age (years) mean (SD)	Gender % female
			2) Control group			
Burns et al., [29]	USA	This RCT tested two 24- month primary care interventions to alleviate the psychological distress of carers of people with AD. The interventions, using targeted educational materials, were a) patient behaviour management only (behaviour care) (A), and b) A + carer stress–coping management.	1) Enhanced care	82 (52.4 %)	65.1 (12.6)	87.4 %
	PC sites		2) Behaviour management	82 (56.5 %)	64.5 (13.0)	84.6 %
Nichols et al., [30]	USA	This clinical translation was developed to test/demonstrate that a proven behavioural intervention for carers of PWD (Belle et al., [33]) could be successfully translated into clinical practice with different types of staff delivering the 6-month REACH VA intervention. This included education, support, and skills training to address five caregiving risk areas: safety, social support, problem behaviours, depression, and carer health. There was no control group.	1) REACH VA intervention	127 (22.8 %)	71.6 (11.6)	92.7 %
	Home-based PC programs		n.a.			
Fortinsky et al., [31]	USA	This quasi-experimental study investigated the value of employing a nurse practitioner with geropsychiatric expertise to augment care from primary care physicians for PWD and their family carers. The intervention was called PPDC. Control group patients and carers received usual care supplemented by educational materials.	1) PPDC program	21 (23.2 %)	67.4 (13.8)	48.0 %
	Community-based PCP group practice		2) Usual care	10 (n.r.)	69.9 (14.9)	70.0 %
Rodriguez-Sanchez et al., [32]	Spain	In a primary health care context, this multicentre RCT tested the effect of a cognitive behavioural intervention developed to improve the mental health of carers of PWD. The control group received usual care.	1) Cognitive-behavioural intervention	83 (7.2 %)	61.1 (11.9)	73.5 %
	PHC centres		2) Usual care	42 (19.0 %)	649 (11.8)	76.2 %

*AD* Alzheimer’s disease, *PC* Primary care, *PPDC* Proactive Primary Dementia Care, *RCT*randomized controlled trial, *REACH VA* Enhancing Alzheimer’s Caregiver Health (Department of Veterans Affairs), *PWD* people with dementia

Three papers originated from the USA [29–31] with one from Spain [32]. They were published between 2003 and 2014. The settings were community and primary care facilities [29–32]. Only one study was a home based primary care program [30].

### Methodological quality

Two out of the four included studies were randomised controlled trials (RCT) using pre and post intervention measures [29, 32]. One study [31] used a ‘non-equivalent control group trial’. In this quasi-experimental trial, a pretest-posttest controlled design was used. Here the assignment to intervention and control groups was non-random and, as a result, the groups may have been different prior to the study (i.e. non-equivalent control group trial). This study compared the intervention with the comparison group using three-point time measures (baseline; 6 months and 12 months after baseline). The remaining study [30] was described as a clinical translation of the REACH II RCT [33] but could also be described as an uncontrolled before and after study. There was no control group but there was comparison between baseline and 6-month follow-up.

The quality ratings of the included studies ranged from 58 % [30] to 93 % [29], with two studies rated as good quality (85 % or more), as illustrated in Table 3. A clear study question was stated in all studies. Furthermore, all studies reported their findings in detail and formulated appropriate conclusions. Baseline and or demographic information were clearly provided except in Nichols et al. [30]. Whilst the attrition rate was reported in all studies, intention-to-treat analysis was only used by Fortinsky et al., [31] and Rodriguez-Sanchez et al. [32]. The most important flaw identified within the included studies related to the lack of participant/investigator blinding with only Burns et al. [29] reporting that researchers involved in the study were blinded to intervention assignment. However, findings from this study are likely to be distorted by ‘contamination bias’ (carers in the control group were exposed to part of the intervention) [34].

**Table 3:** Quality assessment of the selected studies

		Reference			
Quality Item [28]		Burns et al., [29]	Nichols et al., [30]	Fortinsky et al., [31]	Rodriguez-Sanchez et al., [32]
1	Question / objective sufficiently described?	Yes	Yes	Yes	Yes
2	Study design evident and appropriate?	Yes	Yes	Yes	Yes
3	Method of subject/comparison group selection or source of information/input variables described and appropriate?	Yes	Yes	Partial	Yes
4	Subject (and comparison group, if applicable) characteristics sufficiently described?	Yes	Yes	Partial	Yes
5	If interventional and random allocation was possible, was it described?	Yes	N/A	N/A	Yes
6	If interventional and blinding of investigators was possible, was it reported?	Yes	Partial	N/A	No
7	If interventional and blinding of subjects was possible, was it reported?	Partial	No	N/A	No
8	Outcome and (if applicable) exposure measure(s) well defined and robust to measurement / misclassification bias? Means of assessment reported?	Yes	Partial	No	Yes
9	Sample size appropriate?	Yes	Yes	Partial	Yes
10	Analytic methods described/justified and appropriate?	Yes	Partial	Partial	Yes
11	Some estimate of variance is reported for the main results?	Yes	Yes	Yes	Yes
12	Controlled for confounding?	Partial	Yes	No	Yes
13	Results reported in sufficient detail?	Yes	Yes	Yes	Yes
14	Conclusions supported by the results?	Yes	Yes	Yes	Yes
Overall quality score		93%	58%	81%	86%

*N/A* not applicable

### Outcome measures

The included studies investigated the effects of the interventions upon a number of carer outcomes including depression and or depressive symptoms [29–32]; caregiving difficulties and frustrations [29, 30, 32]; carer knowledge of dementia [29–31]; burden [30–32]; time spent providing care [30]; social support [30]; quality of life [32]; well-being [29]; physical health [30] and mental health [32]. These outcomes were measured in a variety of ways making comparison of the findings difficult (Table 4). For instance, two of the three studies [30, 32] reporting on carer burden measured it with the Zarit Burden Interview [35], while Fortinsky et al. [31] measured it with the Short Zarit Burden Interview [36].

**Table 4:** Findings reported in included studies

Reference	Type of study	Outcomes measures		Effect
	Quality score	Outcome	Measurement tool	
Burns et al., [29]	RCT	Affect	CES-D	Significant positive changes in the CES-D over time (*p* = 0.007), with no significant differences between the intervention and control groups (*p* = 0.311)
		Response to the behavioural manifestations of the disease	RMBPC	Significant positive changes in RMBPC scores over time (*p* = 0.010), with no significant differences between the intervention and control groups (*p* = 0.976)
	93 %	Risk of depression	CES-D >16	No significant effects in the proportion of carers with scores of CES-D ≥ 16 (i.e. at risk of depression) between the intervention and control groups
		Well-being	Modified GWB Scale	Significant positive changes in the GWB over time (*p* =0.004) between the intervention and control groups
Nichols et al., [30]	Clinical translation (uncontrolled before and after study)	Burden	Zarit Burden Interview	No significant effects (*p* > 0.05)
		Bother with behaviours	RAM	Significant positive changes in the burden over time (*p* =0.004)
		Caregiving difficulties		No significant effects (*p* > 0.05)
		Caregiving frustrations		Significant positive changes over time (*p* = 0.004)
		Depression	Patient Health Questionnaire	Significant positive changes over time (*p* = 0.009)
	67 %	Impact of depression	RAM	Significant positive changes over time (*p* = 0.01)
		Health behaviours		No significant effects (*p* > 0.05)
		Health status	Medical Outcomes Study Short-Form 36	
		Self-Care/safety	RAM	
		Social Support		
		Hours on duty,		
		Hours providing care		
Fortinsky et al., [31]	Non-equivalent control group	Burden	Short Zarit Burden Interview	No statistically significant changes between the intervention and control groups in any of the median outcome measure scores over time (*p* > 0.05)
		Community support service use self-efficacy	Likert type 10-point score questionnaire	
	68 %	Depressive symptoms	CES-D	No statistically significant changes between the intervention and control groups in any outcome measures after adjusting for the three time points (*p* > 0.05)
		Symptom management self-efficacy	Likert type 10-point score questionnaire	
Rodriguez-Sanchez et al., [32]	RCT	Burden	Zarit Burden Interview	No significant effects for the intervention group compared with the control group (*p* > 0.05)
		Mental health	GHQ -12	A significant reduction in GHQ-12 score for the intervention group compared with the control group (*p* = 0.01)
	89 %	Dysfunctional thoughts about caregiving	Losada questionnaire	Significant positive changes for the intervention group compared with the control (*p* = 0.01)
		Quality of life	Ruiz and Baca questionnaire	No significant effects for the intervention group compared with the control group

*GP* General practice, *PC* Primary care, *PHC* Primary Health Care, *PPDC* Proactive Primary Dementia Care, *RCT* Randomized Controlled Trial

*CES-D* Center for Epidemiological Studies Depression scale, CES-D >16; GHQ-12: General Health Questionnaire; *GWB* General Well-Being scale; Patient Health Questionnaire; *RAM* Risk appraisal measure questionnaire; *RMBPC* Revised Memory and Behavior Problems Checklist

Likert type 10-point score questionnaire (Created specifically for the project)

A summary of findings showing the impact of the interventions on specific outcomes for each included study is provided in Table 4. There is evidence that interventions in general practice settings consistently produce positive benefits for carers of people with dementia in terms of improved psychological well-being, burden, and depressive symptoms. For example, the primary care educational-intervention investigated in Burns et al. [29] is likely to be effective in reducing carer distress and burden in the management of the person with dementia (by increasing carer ability to manage problem behaviours and therefore increasing their competency and confidence) [29]. The community-based intervention (Proactive Primary Dementia Care – PPDC) examined by Fortinsky et al. [31] did not measurably improve burden, community support service use self-efficacy, depressive symptoms, and symptom management self-efficacy in people with dementia or their carers compared to those in the control group [31]. These authors included an education, a support and skills training component to address five caregiving risk areas: safety, social support, problem behaviours, depression, and carer health. However, following the implementation of the intervention in the REACH VA study [30] carers reported significantly improved outcomes including burden, depression, impact of depression on daily life, and caregiving frustrations. The cognitive behavioural intervention investigated by Rodriguez-Sanchez et al. [32] appears promising. Following the intervention, the carers reported significant improvements in their mental health which appeared to have its effect by reducing their dysfunctional thoughts.

## Discussion

To the best of our knowledge this is the first review identifying, appraising and summarising the literature relating to support for carers of people with dementia in general practice. PRISMA standards were adopted making the search strategy extensive, rigorous and reproducible. The four included studies were international and between them included many participants potentially giving these overall findings more weight. Study design varied but all investigated psychosocial interventions for carers of people with dementia with none focussing on carers of stroke survivors. The insights gained from these studies may help to set future research and service evluation agendas.

The interventions identified here were intended to improve carer emotional health, carer knowledge of dementia, caregiving competence and to reduce carer burden, difficulties and frustrations. They are therefore similar in content and intended outcome to the interventions offered in other community settings and likewise the evidence for their effectiveness is mixed [4, 17, 18]. In the context of general practice, our evidence suggests that the implementation of psychosocial interventions may improve well-being and mental health by improving carers’ ability to cope with the behavioural manifestations of the disease and their dysfunctional thoughts about caregiving [29, 32]. Overall the evidence for the impact for these interventions should be treated with caution for three reasons. Firstly, when looking at the effects on burden, quality of life and health status, the impact was not statistically significant. Secondly, when looking at depression, knowledge of illness and caregiving competence, the reported effects were contradictory across studies. Finally, although Nichols et al. [30] reported a significant effect on caregiving frustration, this study scored poorly on methodological quality. Furthermore, the diversity of the outcomes and in the measurement tools used made cross-study comparison difficult.

Earlier reviews [13, 37] also concluded that psychosocial interventions for carers of people with dementia can reduce carer burden. Similarly, our review suggests that psychosocial interventions provided in general practice can have a positive impact on burden, although the findings here were not statistically significant [31, 32]. Only Nichols et al. [30] reported a statistically significant improvement in burden.

Despite its frequent use as an outcome measure, the concept of carer burden is increasingly being questioned for lack of conceptual clarity and definition [38]. Our findings support those of Acton and Kang [39] who showed in their meta-analysis that only multifaceted interventions significantly reduced carer burden. They suggest that carer burden is such a broad concept that interventions may not consistently have any impact on it.

The fact that no studies were found investigating supportive interventions for carers of stroke survivors is surprising given the evidence that these carers regard general practice as an important source of support [8, 40]. This suggests that general practice is possibly either offering little support directed at these carers or that evaluations of the interventions are not being published in peer reviewed journals. Either way, clearly more needs to be known about this given the potentially significant role general practice could play in supporting this important group.

The interventions were mainly intended to alleviate carers’ psychosocial distress and consisted primarily of information and educational materials aimed at helping carers manage the behavioural challenges the person with dementia might display. These interventions focused on providing information to carers about the progression or manifestation of the disease and its management, whilst some interventions also aimed to address personal needs by providing support, skill training, and problem-solving. However, these publications did not always provide sufficient information to allow replication of the intervention and with the exception of Nichols et al. [30], the theoretical basis was not always clear. Furthermore, the evaluations did not provide enough detail to allow identification of the ideal timing of the interventions in relation to the stage of dementia. This is important given the dynamic nature of caring and the often downward trajectory in dementia caring [12].

The vast majority of the carer participants here were female. This finding has been reported elsewhere [8, 41]. However, although female carers generally outnumber male carers [42] they are over-represented in carer intervention studies [43] which may be a significant issue given the evidence that male and female carers often describe different challenges. For example, male carers report less burden than female carers [44] and are also more likely to adopt task-orientated than emotion-focussed strategies [45]. This suggests that male and female carers are are likely to require different types of supportive interventions [46]. Future research should therefore address this to ensure that the interventions are appropriate for both male and female carers.

However, there are also some limitations of the review. Firstly, very few studies were identified limiting our potential conclusions. In order to ensure specificity of the review we did not include studies where the interventions were provided by professionals working outside general practice. These are listed in Additional file 1 but include, for example, interventions provided by social workers [47] or volunteers [41]. All the included studies were written in English, situated in the Western world and therefore potentially excluded some important cultural differences in the outcomes. Family caring has strong cultural influences [48] suggesting that interventions in one cultural group may not be suitable for other cultural groups. Publication bias is another concern in that studies with significant findings are more likely to be published and the dearth of qualitative studies may reflect this bias. The overall quality and the generalis ability of the included studies were variable, so the findings of the present research should be interpreted with caution. Finally we were also unable to find any studies investigating the efficacy of interventions for carers of people living with stroke and future reviews should address this by searching the grey literature.

## Conclusions

In order to understand better the effect of interventions based in general practice, future research should perhaps also adopt mixed methods approaches which should make it possible to explore, for example, intervention acceptability. A health economic perspective to improve understanding of the cost-effectiveness of these varied interventions and programmes would also be an important addition. Further research is also needed to investigate and to clarify the timing and or the support needs carers of stroke survivors [49]. Finally, given the mixed findings of the effectiveness of these interventions for carers in both the general practice and more widely, greater user involvement in developing such interventions may be one means of improving their acceptability, with an impact on attrition and an increased chance of them benefitting both carers and ultimately those they care for.

## Additional file

Additional file 1:
**Studies excluded in the last stage of the screening process.** (DOCX 25 kb)
