# Supplementary material for: General practice based psychosocial interventions for supporting carers of people with dementia or stroke: a systematic review
Source: BMC Fam Pract. 2016 Jan 15;17:3. doi: 10.1186/s12875-015-0399-2 (PMC4714487; doi:10.1186/s12875-015-0399-2)
Supplement: Additional file 1: — Studies excluded in the last stage of the screening process. (DOCX 25 kb) [file 12875_2015_399_MOESM1_ESM.docx]

**Additional file 1** Studies excluded in the last stage of the screening process.

| **References of excluded studies -** *Reason for exclusion* |
| --- |
| *Inappropriate intervention, not general practice based (n=10)*   1. Bass DM, Judge KS, Snow AL, et al. Caregiver outcomes of partners in dementia care: effect of a care coordination program for veterans with dementia and their family members and friends. J Am Geriatr Soc. 2013;61(8):1377-86. 2. Coon DW, Thompson L, Steffen A, et al. Anger and depression management: psychoeducational skill training interventions for women caregivers of a relative with dementia. Gerontologist. 2003;43(5):678-89. 3. Egan M, Anderson S, McTaggart J. Community navigation for stroke survivors and their care partners: description and evaluation. Top Stroke Rehabil. 2010;17(3):183-90. 4. Fortinsky RH, Unson CG and Garcia RI. Helping family caregivers by linking primary care physicians with community-based dementia care services: The Alzheimer's Service Coordination Program. Dementia, Int J Soc Res Prac. 2002; 1: 227 - 240. 5. Fortinsky RH, Kulldorff M, Kleppinger A, et al. Dementia care consultation for family caregivers: collaborative model linking an Alzheimer's association chapter with primary care physicians. Aging Ment Health. 2009;13(2):162-70. 6. Harrington R, Taylor G, Hollinghurst S, et al. A community-based exercise and education scheme for stroke survivors: a randomized controlled trial and economic evaluation. Clin Rehabil. 2010 Jan;24(1):3-15. 7. Jansen AP, van Hout HP, Nijpels G, et al. Effectiveness of case management among older adults with early symptoms of dementia and their primary informal caregivers: a randomized clinical trial. Int J Nurs Stud. 2011;48(8):933-43. 8. Joling KJ, Bosmans JE, van Marwijk HW, et al. The cost-effectiveness of a family meetings intervention to prevent depression and anxiety in family caregivers of patients with dementia: a randomized trial. Trials. 2013;14:305. 9. Phung KT, Waldorff FB, Buss DV, et al. A three-year follow-up on the efficacy of psychosocial interventions for patients with mild dementia and their caregivers: the multicentre, rater-blinded, randomised Danish Alzheimer Intervention Study (DAISY). BMJ Open. 2013;3(11):e003584. 10. Yordi C, DuNah R, Bostrom A, et al. Caregiver supports: outcomes from the Medicare Alzheimer's disease demonstration. Health Care Financ Rev. 1997;19(2):97-117. |
| *Inappropriate study population (n=9)*   1. Callahan CM, Boustani MA, Unverzagt FW, et al. Effectiveness of collaborative care for older adults with Alzheimer disease in primary care: a randomized controlled trial. JAMA. 2006;295(18):2148-57. 2. Cherry DL, Vickrey BG, Schwankovsky L, et al. Interventions to improve quality of care: the Kaiser Permanente-alzheimer's Association Dementia Care Project. Am J Manag Care. 2004;10(8):553-60. 3. Donath C, Grässel E, Grossfeld-Schmitz M, et al. Effects of general practitioner training and family support services on the care of home-dwelling dementia patients--results of a controlled cluster-randomized study. BMC Health Serv Res. 2010;10:314. 4. Downs M, Turner S, Bryans M, et al. Effectiveness of educational interventions in improving detection and management of dementia in primary care: cluster randomised controlled study. BMJ. 2006;332(7543):692-6. 5. Eichler T, Thyrian JR, Fredrich D, et al. The benefits of implementing a computerized intervention-management-system (IMS) on delivering integrated dementia care in the primary care setting. Int Psychogeriatr. 2014;26(8):1377-85. 6. Lee L, Hillier LM, Stolee P, et al. Enhancing dementia care: a primary care-based memory clinic. J Am Geriatr Soc. 2010;58(11):2197-204. 7. Menn P, Holle R, Kunz S, et al. Dementia care in the general practice setting: a cluster randomized trial on the effectiveness and cost impact of three management strategies. Value Health. 2012;15(6):851-9. 8. Rondeau V, Allain H, Bakchine S, et al. General practice-based intervention for suspecting and detecting dementia in France: a cluster randomized controlled trial. Dementia 2008;7(4):433-450. 9. Simon C, Kumar S, Kendrick T. Formal support of stroke survivors and their informal carers in the community: a cohort study. Health Soc Care Community. 2008;16(6):582-92. |
| *Inappropriate study design (n=3)*   1. Austrom MG, Hartwell C, Moore P, Perkins AJ, Damush T, Unverzagt FW, Boustani M, Hendrie HC, Callahan CM. An integrated model of comprehensive care for people with Alzheimer’s disease and their caregivers in a primary care setting. Dementia. 2006;5(3):339–352. 2. Austrom MG, Damush TM, Hartwell CW, et al. Development and implementation of nonpharmacologic protocols for the management of patients with Alzheimer's disease and their families in a multiracial primary care setting. Gerontologist. 2004;44(4):548-53. 3. Meeuwsen EJ, German P, Melis RJ, et al. Cost-effectiveness of post-diagnosis treatment in dementia coordinated by Multidisciplinary Memory Clinics in comparison to treatment coordinated by general practitioners: an example of a pragmatic trial. J Nutr Health Aging. 2009;13(3):242-8. |
| *Not report empirical findings (n=2)*   1. Edwards R, Voss S, Iliffe S. Education about dementia in primary care: is person-centredness the key? Dementia (London). 2014;13(1):111-9. 2. Keady J, Nolan M. A stitch in time. Facilitating proactive interventions with dementia caregivers: the role of community practitioners. J Psychiatr Ment Health Nurs. 1995;2(1):33-4 |
| *Inappropriate intervention – pharmacological*   1. Meeuwsen EJ, Melis RJ, Van Der Aa GC, et al. Effectiveness of dementia follow-up care by memory clinics or general practitioners: randomised controlled trial. BMJ. 2012;344:e3086. |
